# Gender and agricultural innovation in Oromia region, Ethiopia: from innovator to tempered radical

**DOI:** 10.1080/09718524.2018.1557315

**Published:** 2019-02-05

**Authors:** Cathy Rozel Farnworth, Diana E. López, Lone Badstue, Mahelet Hailemariam, Bekele G. Abeyo

**Affiliations:** a Pandia Consulting, Münster, Germany;; b Knowledge, Technology & Innovation, Wageningen University & Research, Wageningen, Netherlands;; c International Maize and Wheat Improvement Center (CIMMYT), México-Veracruz, Mexico;; d Women at Work, Addis Ababa, Ethiopia;; e CIMMYT Ethiopia, Addis Ababa, Ethiopia

**Keywords:** Tempered radicals, agricultural innovation processes, gender, wheat, Ethiopia, GENNOVATE

## Abstract

Tempered radicals are change agents who experience the dominant culture as a violation of the integrity and authenticity of their personal values and beliefs. They seek to move forward whilst challenging the status quo. Does the concept provide a useful analytic lens through which the strategies of women and men farmer innovators, who are ‘doing things differently’ in agriculture, can be interpreted? What are their strategies for turning ambivalence and tension to their advantage? The paper uses research data derived from two wheat-growing communities in Oromia Region, Ethiopia, an area characterized by generally restrictive gendered norms and a technology transfer extension system. The findings demonstrate that women and men innovators actively interrogate and contest gender norms and extension narratives. Whilst both women and men innovators face considerable challenges, women, in particular, are precariously located ‘outsiders within,’ negotiating carefully between norm and sanction. Although the findings are drawn from a small sample, they have implications for interventions aiming to support agricultural innovation processes which support women’s, as well as men’s, innovatory practice. The framework facilitates a useful understanding of how farmer innovators operate and in particular, significant differences in how women and men interrogate, negotiate and align themselves with competing narratives.

## Introduction

This paper applies a ‘tempered radicals’ perspective to agricultural innovators in two communities in Oromia Region, Ethiopia. This intriguing concept was developed to describe individuals who experience the dominant culture of their organization as a violation of the integrity and authenticity of their personal values and beliefs. They seek advancement whilst criticizing the status quo and seeking to transform it (Meyerson & Scully, 1995). Tempered radicals express three characteristics. First, they are moderates rather than firebrands. Moderation is necessary since they cannot risk being isolated or cast out of their organizations, or, in our study, their communities since this could harm them. The question then arises: how do tempered radicals manage their status of ‘outsiders within’ (Meyerson & Scully, 1995, p. 589). What are their strategies for innovating yet belonging? Second, tempered radicals have to be tough, like tempered steel. They need to be resilient and persistent in their efforts to open up spaces for innovation in the face of resistance and powers trying to force compliance. Third, they must have a temper. This can be defined as both an ‘outburst of rage’ and ‘composure’ (Meyerson & Scully, 1995, p. 586). Temper is an expression of agency (defined as the ability to set a goal and act upon it) and a motive force for change.

Meyerson and Scully do not draw on a distinct body of literature to develop the concept of tempered radicals; rather, the concept emerged from the authors’ personal experiences and informal conversations with people who do not fit neatly with their organization’s culture. They defend their subjectivity as pushing the boundaries of social science writing and as drawing inspiration from feminist scholarship (Meyerson & Scully, 1995, p. 587). In their paper, shared subjective experiences coalesce to permit the conceptualization of a new kind of change agent. Meyerson and Scully explain (Meyerson & Scully, 1995, p. 586): ‘We chose the name “tempered radical” deliberately to describe our protagonist. These individuals can be called “radicals” because they challenge the status quo, both through their intentional acts and also just by being who they are, people who do not fit perfectly.’

This focus on individuals emerging as protagonists of change, rather than examining carefully planned organizational strategies or trained change agents, has provided scope for the concept to be tested in a variety of settings, including the public sector (for example, Alston, 2005; Carlone, Haun-Frank, & Kimmel, 2010) and in the private sector (for instance, Kirton, Greene, & Dean, 2007). These studies explore tensions and interactions between individuals and the various structures they wish to change. Walton and Kirkwood (2013) examine how environmental entrepreneurs in New Zealand operate businesses. They seek to reconcile potentially opposing goals: maintaining commitment to environmental objectives whilst making money. The study concludes that although ecopreneurs challenge business norms their ability to create positive environmental impact is limited due to the weak structural position of their businesses in complex value chains. Farnworth, Jiggins, and Gurung, (2007) conducted discussions with scientists in agricultural research organizations. They concluded that tempered radicals in these settings develop two typical change strategies. One involves deliberate efforts to create, and draw personal strength from, social relationships within and across the boundaries of the organization. The second involves developing professional practice as a means of eliciting a different kind of performance from the organization (Farnworth et al., 2007).

This paper considers whether the concept of tempered radicals, which was developed to interrogate how individuals try to initiate change processes in organizations, can have validity in rural agricultural settings. Our hypothesis is that this concept provides a useful lens through which the strategies of women and men farmers innovators – recognized by community members as people who ‘do things differently’ – can be captured and analyzed. In particular, we explore the degree to which the concept allows understandings of interactions between individual agency and locally relevant cultural norms – which operate to provide bounded sets of rules to live by – to be developed. We further hypothesize that women and men may use broadly similar strategies, but that the implications for their personal standing in their communities, and the costs and benefits of using these strategies, may be different.

To examine these hypotheses the paper draws on Ethiopian research data developed through GENNOVATE, a research study across 137 rural communities in 26 countries in Asia, Africa, and Latin America – including eight in Ethiopia (Badstue et al., 2018; Petesch et al., 2018). The analysis provided here draws initially on research conducted with 136 women and men respondents in two study sites in Oromia Region in 2016 before turning to focus on eight individual innovators in the same sites, four women and four men. The small sample size is consistent with other papers cited above on tempered radicals as well as with literature which highlights the value of focusing on few individuals to investigate their views, beliefs, and strategies in greater depth (Alshenqeeti 2014; Kvale & Brinkmann, 2009). We decided to apply the concept to innovators in Oromia Region because the subjective experiences and stories of the innovators, as recorded by the research teams, provided particularly rich and complex data which invited further analysis.

Context is of course important. We provide a review of gender norms in Oromia and an overview of extension services – in Ethiopia in general and as discussed by the 136 respondents in the study sites. We also profile the two study sites. We expect this to provide a rich understanding of the environment within which the selected innovators operate. In GENNOVATE, and in this paper, the word technology is used to describe ‘things’ such as improved wheat varieties, agronomic practices, and the use of mechanical equipment. The term innovators is used to describe people doing something different, or new, for them – rather than to novelty in absolute terms (Badstue et al., 2018).

The analysis is expected to provide a contribution to broader and emerging thinking on agricultural innovation processes in similar contexts, in particular to farmer-driven innovation (Röling, 2009). The contextual embeddedness, complexity and multi-leveled, inter-meshed and evolving nature of innovation processes has been emphasized by several authors (Geels, 2011; Hall, 2007; Klerkx, van Mierlo, & Leeuwis, 2012; Leeuwis, 2013; Schut, van Paassen, Leeuwis, & Klerkx, 2014; Schut et al., 2016). Much of the literature on agricultural innovation focuses on processes and systems, and can often appear to lose sight of the people in those systems. Alsos, Ljunggren, and Hytti (2013) note this and add, ‘When people are not visible in the discourse, gender easily becomes invisible’ (p. 3).

Research shows that both structural and individual factors influence women’s and men’s engagement with innovation (Ahl, 2006; Ahl & Marlow, 2012; Pecis, 2016). A number of authors explore the dialectical relations between gender and innovation (see Badstue et al., 2018, Bossenbroek & Zwarteveen, 2014; Drew, 2014; Padmanabhan, 2002; Pyburn, 2014). Kingiri (2013) concludes that capacity to innovate is determined by a combination of individual skills, actions, and experiences as well as by broader institutional, market, policy, and financial domains. Fostering gender-inclusive innovation processes demands investment in both the capacities and empowerment of individual farmers as well as structural change to the systems they live and work within (Farnworth, Stirling, Chinyophiro, Namakhoma, & Morahan, 2018; Kingiri 2013; Pyburn, 2014).

Douthwaite and Gummert (2010) argue, partly in responses to overly systemic analyses of innovation, that the issue in agriculture, today, is not technological scarcity but rather innovation capacity scarcity. Together with other researchers, Douthwaite and Gummert developed a ‘learning selection’ model which is built on an analogy with Darwinian natural selection. According to this, technologies are developed through a process of novelty generation. Technologies then undergo selection – the mechanism whereby positive traits are retained and detrimental ones rejected – as stakeholders adapt and improve them over time. Finally, beneficial technologies are diffused to other territories. The consequence is that the ‘technology increases in fitness’ (Douthwaite & Gummert 2010; Douthwaite, Keatinge, & Park, 2002). However, their gender-blind analysis raises the concern that they have ignored how gender is embedded in innovation systems and processes in subtle but powerful ways and has important effects on innovation potential – whether or not efforts are made to measure these effects. This paper, therefore, considers whether this rather benign conceptualization of innovation processes has any purchase for tempered radicals in our communities.

Finally, we refract our analysis of tempered radicals through considering their interactions with social institutions. Social institutions comprise the norms and rules of behavior which shape, though do not fully determine, how individuals and organizations behave (Stewart, 2013) and in this, can be considered similar to formal organizations. Social institutions include visible and deep structures. Visible structures have recognizable forms. These include how households (monogamous, polygamous, etc.) and organizations such as producer cooperatives are set up and operate. The term deep structure refers to the underlying values, assumptions, and ideologies that perpetuate, characterize, and inform these visible entities. Deep structures underlie and ‘justify’ how societies are organized, how resources are distributed, and how laws are written. In many agrarian societies, for instance, sons rather than daughters tend to inherit land. This reflects a norm drawn from the deep structure. National legislation in some countries may support this practice through acknowledging customary law. This is a visible expression of deep structure. These underlying norms affect the choices of an individual between the different capabilities – what a person can be – ‘beings,’ and what they can do – ‘doings’ or goals they may seek to pursue (Farnworth et al., 2018; Nussbaum, 2003; Sen, 1985).

Stewart (2013) argues that no-one can experience complete autonomy: their choices are influenced by underlying norms. This paper acknowledges these intrinsic limitations, but is based on the premise that tempered radicals consciously ‘play’ with cultural norms in order to reach their goals (Farnworth et al., 2007). In this way, they may achieve freedoms they value, and in the process potentially change cultural norms.

## Materials and methods

The paper draws upon a sub-set of the GENNOVATE Ethiopian research data drawn from two kebeles in Oromia Region. Kebeles are the smallest administrative districts in Ethiopia and correspond roughly to villages. Following the protocols of GENNOVATE, kebele selection was based on a purposive, maximum diversity sampling approach guided by two dimensions which were considered significant for understanding gender differences in agricultural innovation (i) economic dynamism, and (ii) gender gaps in assets and capacities^1^ (see more details on the Gennovate methodology in Petesch et al., 2018). The focus on these two dimensions responds to findings (see the McKinsey Global Institute Report, 2015, for example) on associations between countries with greater gender equality and higher levels of economic growth. A 2 × 2 matrix with four variables was developed: wide gender gaps or narrow gender gaps on one axis, and high economic dynamism or low economic dynamism on the other. The two kebeles discussed in this study were purposively selected because they fell within the quadrant expressing high economic dynamism and high gender gaps.

The research process involved conversations using five research tools including focus group discussions (FGDs) and semi-structured interviews with 136 farmers (women and men in equal numbers) from poorer to better off sections in the two kebeles in 2016. This was complemented by developing community profiles in each location. Material from these discussions and the community profiles is used to provide local contextual information on innovation processes and thus deepen the findings of the literature review. The material for the tempered radical discussion draws specifically on eight individual ‘innovation trajectory interviews’ which were conducted with two men and two women in each kebele. The selection of innovators – classified as people known in their community for trying out new things in the way they do agriculture – was informed by kebele leaders and other key informants, as well as by women and men who participated in various FGDs. This reliance on diverse local respondents to identify individual innovators was used to reduce selection bias.

The use of interviews in qualitative research is recognized to be ‘a valuable method for exploring the construction and negotiation of meanings in a natural setting’ (Cohen, Manion, & Morison, 2007:29). It allows the researcher to investigate people’s views and beliefs in detail, contextualizes individual experiences, and enables interviewees to ‘speak in their own voice and express their own thoughts and feelings’ (Berg, 2007: 96). Analyzing the findings from interviews with locally recognized innovators provides a close counterpart to the research methodology deployed by Meyerson and Scully (1995) and other researchers, to help identify and characterize tempered radicals. We wanted to see if the way innovators in rural Oromia Region conceptualized themselves and their practice permits us to call them ‘tempered radicals.’

The innovation trajectory interview schedule interrogates how thinking in relation to farming practice has changed over time. What drives people to innovate? How do they select, adopt, and adapt technologies? How do they interact with wider institutional structures in order to push forward their agendas? In what ways do gender norms influence their strategies for change? The interview opens with a five-step ladder called the Ladder of Power and Freedom. Each innovator is asked to rate their current ability to make important decisions in their lives, including about their working life, starting/maintaining a business or an income-generating activity, their use and control of productive resources, and whether to start or end a relationship. The bottom step, Step 1, represents almost no decision-making power. The top step, Step 5, indicates people who are able to make most major life decisions. They are then asked to locate the position they occupied on the ladder 10 years ago, and to reflect upon the reasons for the change. The schedule continues with further set questions to help respondents construct their individual history of innovation. The use of the ladder, and ratings, helps to build a comparative story of how the personal agency is perceived, by the respondent, to have changed over time, and to analyze the reasons why. Ratings also allow some degree of comparison between respondents.

NVivo Qualitative Software was used to conduct the initial variable-oriented analysis. This permitted the identification of emergent themes within and across the data. This was followed by systematic in-depth data analysis. The analysis was respondent-led in that it relied on respondent terminology and framing (Farnworth, 2009).

## Site description

We provide a short portrait of the study sites before discussing research by a number of scholars on gender relations and the extension services in Oromia Region. As part of the latter, we include some of our own findings from the two study sites. Taken together, the three sub-sections provide information on the institutional context within which our tempered radicals seek to innovate.

### Study sites

The two kebeles are close to commercial centers and markets. Diversification of livelihoods and employment opportunities is increasing. Over the past 10 years technologies including the use of agricultural machinery, improved wheat varieties, and other improved as well as novel crops and livestock have been introduced. Available inputs include inorganic fertilizers (DAP, NPS, NPS + B, urea), organic manure, fungicides for wheat rust control, and herbicides. Local flour mills established in the last decade have stimulated demand for locally grown wheat. The institutional environment includes indigenous institutions with wide membership which can be single or mixed sex. Iquib are saving groups. Members access funds in rotation weekly or monthly. Idir are funeral societies to which members contribute monies monthly in order to borrow tents and other equipment during funerals and weddings. Both women and men participate in these institutions though women participate more often. Debo is a reciprocal farming arrangement to help manage large agricultural tasks, including row planting, house construction, etc.

Important cereals, legumes, and oil crops include wheat, barley, oats, maize, and beans, peas, chickpea, and sesame. Improved varieties of wheat, mainly derived from CIMMYT germplasm, have been introduced including: Digalu, Hidase, Huluka, Tusie, Hawi, Mada Wolabu, Danda’a, Kakaba, and Galema. Danda’a and Digalu are the most popular because they are considered to produce high-quality flour and bread. Carrots, beetroot, garlic, cabbage, and onions are widely grown. Livestock includes dairy cattle, oxen, horses, donkeys, sheep, goats, and local poultry. Poultry is unquestionably under women’s control. Women have the right to produce and sell butter made from dairy milk and to sell eggs. Land registration, which began a decade ago, indicates husband and wife as co-owners of the land.

### Gender relations in Oromia region

Gender studies in Oromia Region provide a complex portrait of the position of women. In Ambo District women work more than double the hours worked by men in the peak agricultural season because they conduct or contribute to almost all farming activities as well as homestead activities. However, this work is rarely recognized (Ogato, Boon, & Subramani, 2009a). The sense that women’s contributions are not valued is drawn out in a study of oral narratives among the Jimma Oromo (Alemu, 2007). In such narratives men and ‘masculine attributes’ are valued positively. Conversely, women are portrayed as cruel, sexually rapacious, and as lacking the mental and physical capacity necessary to hold leadership positions. Oral narratives also prescribe gender-appropriate behavior. Women should be obedient, subservient, respectful, and faithful to men, whereas men should be powerful and authoritative. These stories, Alemu argues, play a strong role in maintaining the subordination and marginalization of women. They allow us to ‘assert and maintain our own interests not just by advancing a view of ourselves but also by undermining the views that others advance of themselves’ (Novitz, 1997, p. 146 cited in Alemu, 2007). Storytelling is an example of invisible power (Gaventa, 2006, p. 29). Significant issues are not addressed in decision-making arenas because stories influence, a priori, how individuals think about themselves in the world: their beliefs, sense of self, and degree of acceptance of the status quo.

Debsu (2009) finds discrepancies between stories and what actually happens. For instance, pastoralist Guji-Oromo women have more cultural and economic rights than immediately apparent. Although their oral narratives present women as ineffective, in reality, women act as mediators and peace negotiators during and after conflict. They also enjoy indirect claims to family property under customary laws. The shift from pastoralism to agro-pastoralism, however, is resulting in women becoming subjected to increased domestic and other work burdens. Statutory laws applicable to sedentarized communities are making the position of women worse by eliminating customary economic securities and legal protections.

Hebo and Shigeta (2014) find that women in West Arsi, Oromia are challenging institutional structures. They are starting to speak openly of power imbalances and their desire for change. These narratives are emerging due to new laws and policies at national, regional, and local levels on gender equality. However, local norms constrain their application. Men still take major decisions, women rarely obtain property after divorce, and women’s engagement in transfers of land and livestock remains limited. The ways in which men use established social structures to resist change are clear, but women also confound the potential of change processes by remaining loyal to some traditional norms.

A study of how Arsi Oromo translate concepts of gender equality into their own context helps to explain this ambivalence (Østebø, 2015). Gender experts in the Ethiopian government machinery consider gender equality means sameness, as expressed through equalizing the gender division of labor. However, at the community level, women and men emphasize the importance and the ideal of gender complementarity. By this, they mean collaborative work and mutual agreement through conjugal dialog. Østebø finds that ‘grassroots’ people do not simply assimilate or reject concepts of gender equality promoted by gender experts or development brokers, but are actively engaged in dialog with such discourses. They translate and recreate meanings of gender equality that resonate with local values, needs, and priorities. In particular, local women and men appear to ‘silence’ the version of gender equality that promotes sameness.

Taken together, these case studies articulate emerging tensions between visible and deep structure and how women and men struggle to resolve these tensions.

### Extension environment

Agricultural services in Ethiopia are generally ‘top–down’ despite formal decentralization (Cullen, Tucker, Snyder, Lema, & Duncan, 2014; Hall, 2007; Hall et al., 2010; Ragasa, Berhane, Tadesse, & Taffesse, 2012). Extension services are supply-driven, with off-the-shelf technologies transferred to farmers without expectation of further adaptation by farmers (Ogato, Boon, & Subramani et al., 2009b). The extension is disseminated through model farmers and agricultural extension agents known as development agents (DAs). They are expected to persuade their neighbors to adopt new technologies and are usually selected by kebele leaders – local government officials. Kebele leaders act as intermediaries between the government and the wider community. They are constrained by higher level decision-making processes at regional and national level which they have little power to change and are often inexperienced in stakeholder consultation. This contributes to weak institutional coordination between stakeholders (Cullen et al., 2014).

The institutional context is such that even methodologies which have been developed in response to thinking around innovation as a process rather than an outcome (Röling, 2009) tend to be captured and slotted into the technology transfer model. A study on Innovation Platforms (IPs), for example, revealed that despite their intended purpose as a forum for learning and action involving a group of actors with different backgrounds and interests (Cullen, Tucker, & Homann-Kee Tui, 2013; Swaans et al., 2013), kebele leaders viewed farmers as implementers of technologies rather than co-designers, and they did not see themselves as co-learners alongside farmers. Rather, they complained about farmers’ lack of knowledge, inappropriate farming practices, and short-term vision. Such perceptions undermined farmer self-confidence to the extent that some appeared to internalize the narratives presented by decision-makers. This compromised the effectiveness of the IP (Cullen et al., 2014).

Ethiopian women smallholders are particularly disadvantaged because they have limited access, compared to men, to productive assets including irrigation water, credit, extension services, and rural institutions and thus find it harder to implement innovations (Mulema, Farnworth, & Colverson, 2016; Ogato et al., 2009b). A study using a regionally-representative dataset of 7500 households demonstrated women are much less likely than men to obtain extension services. However, female-headed households with more males in the household and more assets in terms of land and livestock are more likely to be visited or to initiate visits from extension providers. Holding other factors constant, the plots of male and female farmers are equally productive but women’s lower access to quality extension services, radio, inputs, and poorer plot quality explain their lower productivity (Ragasa et al., 2012).

The differences in gendered access to extension agents emerged strongly in the broader discussions involving 136 respondents across the two kebeles. Men farmers mentioned ‘government attention and support,’ ‘follow up by experts,’ ‘success of model farmers,’ and ‘information from experts and the media’ (and money) as key support factors to innovation. Women farmers did not mention any of these factors. Rather, they stated that women in male-headed households are not invited to extension meetings, and that women-headed households are also not invited, though ‘if she has a male child then he goes. But they often do not pass on the information fully. It is difficult to get that information.’ This remark was seconded by a married woman: ‘The extension services do not see women as farmers even though women farm alongside men. The husband comes back home and does not communicate with us. We depend on our own knowledge derived from experience.’

When asked about factors which hamper innovation, men spoke about lack of support from the community, fear of taking risks, lack of money and lack of markets for the innovation. They added that the first to try new technologies are men with extra land and that the extension workers work closely with them. However, information is ‘transferred whenever we meet in social gatherings and community meetings. Whenever anyone wants to know about a technology they can visit the model farmer’s plot.’ Although access to technical information is readily available to men through informal and formal networks they find the stringent technical training offered by extension workers difficult to follow. One man explained, ‘The extension agents did everything systemically and in a precise way. How much seed needed for a plot, how deep it should be tilled, in what way and when to plant and how much and in what way to use fertilizer … it was very complicated and very different to what we are accustomed to.’ Many farmers, therefore, failed to apply the recommendations ‘correctly.’ In fact, few farmers adopt new technologies and practices in the study sites although they observe innovators with keen interest.

Women spoke repeatedly about ‘discouraging words from those people from whom you need support’ and ‘lack of opportunity to do your own thing. If someone else decides for you or if you are waiting for them to decide, then innovation may not happen.’ They also spoke of their lack of knowledge, fear of the jealousy of others, and a simple lack of physical space in which to experiment. They asserted that men model farmers are the first to plant new varieties and to be ‘praised.’ The same respondents remarked that women model farmers, though they are very few, retain ‘the knowledge and the benefit.’ The idea that knowledge was a limited good with quasi-physical properties, hoarded and not shared, was articulated repeatedly. Speaking of other farmers who have adopted new varieties of wheat, one woman said, ‘I am sure they are not obliged to share the information, so why should they?’ The poor women FGD respondents asserted they are excluded from meetings because men refuse to sit with them. This is allegedly because poor women usually have just one item of clothing, which they sleep in, carry children in, and wear when cooking over the open fire.

Women and men discussed in detail the high expense of adopting new seeds and technologies. Many cannot afford to apply fertilizer and seed as recommended, and row planting is a particular challenge due to its labor intensity and lack of locally available and affordable row seeders. Study participants frequently remarked that only unsuitable wheat seed is available, or that seed is not offered on time. When it arrives, farmers with privileged access to information receive it first and ‘poor farmers return empty-handed.’

The data in this section suggests that the innovation environment within which the eight innovators live and work is configured by top-down ‘malestream’ extension narratives. This leads to a form of ‘conceptual lock-in’ (Farnworth & Colverson, 2015) which casts men as farmers and decision-makers. Women are almost entirely excluded by this meta-narrative. Their exclusion is reinforced by deep socio-cultural norms which do not acknowledge women as farmers in their own right.

## Tempered radicals: the findings

Having surveyed the literature on cultural norms and the extension system, and data provided through the wider GENNOVATE respondent profile, we now turn to our eight innovators. We open with a short profile of each person. All names are pseudonyms. We then discuss the findings under four sub-headings: personal innovation history; innovator capacities; supporters of innovation; and benefits (incentives) supporting innovation. We use simple tables to provide a snapshot of key findings and to help orientate the reader at the beginning of each sub-heading. We exclude some material. The innovators discussed assets required for innovation. Unsurprising, these included land, labor and finance and are not discussed further here.

The men innovators

- Daniel is 52, married, and has 10 children. He has completed primary education and owns 2 ha land. He has 5 cattle/oxen, a dual ox-drawn plow, 10 small ruminants, and 7 chickens.
- Ibrahim is 54 and married. Like Daniel, he has 10 children and has completed primary schooling. He owns 2 ha land, has 4 cattle/oxen, 7 small ruminants and 10 poultry.
- Soloman is 50, married, has six children and did not complete primary schooling. He has 2 ha land, two oxen, one horse, three heifers, five chickens, and a dual ox-drawn plow.
- Yonas is the youngest at 40. He is married with six children and did not complete primary education. He owns just 0.25 ha (called a timad), one cow, and a dual ox-drawn plow.

The woman innovators

- Hannah is 39 and a widow with five children. She completed primary education and owns 1 ha land. She has 2 oxen, 1 cow, 6 sheep, 21 hens, and a cockerel.
- Martha is 45, divorced and has four children. She did not go to school. She owns 0.25 ha, 30 hens, and 3 cockerels.
- Aysha is 55, divorced and has nine children. She did not attend school. She owns 2 ha and one ox, one cow, one heifer, two sheep, six poultry and a sickle. She is a model farmer.
- Mimi is 44, has six children, and is the only married woman of the four. Her husband is not a farmer. She did not complete primary school. She owns 1 ha arable land and 1 ha pasture, two oxen, cows, and horses, two chickens, one beehive and a cart.

### Personal history of innovation

Each innovator was asked to place themselves on the Ladder of Power and Freedom today and 10 years ago, and to explain the reasons for their rating. All respondents felt their lives have improved. However, there were gender differences in their testimonies. Men did not recount personal stories in their explanations. This is to sharp contrast to women who provided detailed life histories to explain how they had become innovators. Three out of four women interviewed related their improved placing to the fact that over this time period they had become widowed or divorced. Hannah, for instance, explained that, when married, she was on step 1. After being widowed she slowly moved up to 4. Today, she says, ‘Recently, though, things have become a struggle. Between the time my husband died and my son grew up I would rate my decision power as 5 but now I am at 4 as my son is interfering so much.’

Martha explained her step up the ladder from 3 to 5: ‘I make my plans myself. I think over what I want to do. I never wait for any one’s permission. I may consult them but the final decision is mine. Everybody knows where I was. I was married 10 years ago. I used to buy and sell wheat. This created a lot of problems. My husband was drinking and beating me. I could not stand it anymore, so I asked for divorce. Since then no one has ever decided for me.’ The rest of her story details on-going marginalization and outsider status; her sense of strong personal agency is not reflected in wider acceptance in the community.

Men talked about their move up the ladder very differently. All discussed the rapid pace of change in society and the consequent need to adjust. Daniel commented, ‘Technology has expanded, access to technologies is better, and with this life has also changed. I heard about the new varieties three years ago. I started immediately.’ Yonas and Ibrahim voiced concerns related to the increasing cost of living. Yonas explained that ‘To cope with difficult situations you need to decide fast, and decisively.’

Men, regardless of land size, express a sense of ‘power within’ and intellectualize the forces of change. Women provide deeply personal interpretations of their lives as ‘power won,’ recognizing that this power is continually challenged. In both cases, however, women and men articulate dissonance between their present lives, their community, and broader processes of change.

### Innovator capacities

We asked our innovators to define capacities, using their own words, which they consider essential to innovate. Their definitions are summarized in Table 1. The X in each column refers to the number of innovators who discussed a particular capacity. For example, all women consider curiosity important; three men do. We discuss each capacity below.

**Table 1.** Capacities of innovators.

	Women	Men
Personal curiosity/freedom of mind	XXXX	XXX
Ability to learn from mistakes	XXX	X
Positive attitude to risk	XXX	
Skills/knowledge	X	XXX
Willingness to work hard	X	
Technology adaptation	XX	XX

#### Curiosity

Aysha and Hannah consider curiosity – which appears to be broadly defined by respondents as an eagerness to learn, and to do, something new – the starting point of innovation and the core impulse for change. Hannah asserted that ‘freedom of mind, observation, trying hard to work and prepare your mind and body to work and be fruitful’ is central to her innovative practice. Daniel remarked, ‘I always want to change my life to see new things.’ Ibrahim confessed how, even in bed, he is always making plans. The more he succeeds the more his ‘enthusiasm and appetite for work open up.’

#### Learning from mistakes

The four women openly discussed their failures and the importance of feeling able to start over. Hannah described how over 2 years she failed to obtain a good wheat harvest, no more than 20 quintals^2^. However, ‘I enjoyed the time I spent trying to understand what I could do better. I was proud of myself. I was happy because I experimented and managed to get 41 quintals of wheat. That was the best time I had.’ Just one man, Ibrahim, talked about the importance of learning from one’s mistakes. He ascribed his success to this.

#### Positive attitude to risk

Only women discussed risk. They saw it as an intrinsic element of moving forward and achieving a better quality of life. Mimi stated bluntly that since the land is hers it is for her to take the risk, but other women explored their feelings around risk. Martha said, ‘I am the kind of person who is not afraid. I made a mistake this year, but next year I will correct that mistake.’ Hannah considered how ‘fear pulls you back so you need courage. Taking risks can cost you a lot. If I do not have a fallback plan it could be very hard.’ At the same time, she was ready, because the decision is hers, to ‘take responsibility’ for success or failure.

#### Skills and knowledge

Men and women respondents argued that innovation must build on – to use their terminology – ‘basic farming skills.’ This appears to refer to a set of agricultural competencies won over many years which permits skill-holders to be moderately successful at farming. It is upon this basis that the crucial ingredient of knowledge – by which men mean technical knowledge acquired through the extension services – followed by experimentation with that technical knowledge – must be added to allow innovation to occur. For instance, Soloman spoke of how he previously ‘farmed on impulse.’ He denigrated farmers who continue to do so and considers he has changed totally: ‘The way I plow the land, the way I plant, the way I apply fertilizer and pesticide is completely different. Even the quantity of seed we use in a plot is calculated according to the size of the land as the DAs recommend. They told us that we have to use a specific amount of seed for 0.25 ha. The timing and use of fertilizer and pesticide should be clear. All this requires knowledge and experimentation.’ In other words, male innovators identified knowledge as deriving primarily from an outside source (the extension services) and felt they must adhere to technical guidelines. Deviance through experimentation is permissible provided the guidelines have first been fully understood and applied. Yonas spoke of how his encounters with new technologies have opened his mind, and said, ‘No one is dropping out once they have started. There is no turning back to the traditional practices.’

Hannah – the only woman to use the term knowledge – defined knowledge differently, seeing it as emerging from reflection on her past practice. For her, knowledge combines learning from the past, forecasting activities to be done in the future, securing appropriate training, and calculating expenditure carefully.

#### Hard work

Only women talked of the need to work hard. They have limited ability to call upon the labor of others and thus conduct almost all the work themselves. Hannah and Martha work as daily laborers on other farms, make handicrafts, and buy and resell vegetables and wheat. Hannah recounted the costs. ‘I don’t eat or sleep on time. I work on some things at night. My social life has been spoiled. I do not have close friends. I do not drink coffee with neighbors. I work and work.’ Martha describes a similar life. ‘I never pile up work for tomorrow or next week. I try to accomplish everything now and today. So I am tired all the time. But I am ok. I am healthy.’

#### Technology Adaptation

Women and men talked in depth about experimenting with the Broad Bed Maker (BBM), a farming technology introduced by the extension authorities. This is a simple, locally manufactured farm implement that is attached to the plow and is pulled by a pair of oxen. The plow makes furrows while the two wings of the BBM scoop the soil towards the middle to make a raised bed about 80 cm wide and 15 cm high. The two furrows located on either side of the bed serve as outlets for excess water thereby improving soil drainage. This allows early planting of wheat, followed by planting of a second crop of legumes on the residual moisture immediately after harvesting the wheat.

Both women and men respondents have innovated around the BBM. Hannah, for instance, could not afford to buy one and no one would lend her one. So ‘I decided to build one for myself from wood and it has worked well for the past year.’ Soloman improved on its design. ‘When I tried it, I noticed that because it is made of steel it is too heavy for the oxen. I immediately set out to improvise on it by replacing the steel with wood to make it easier for the oxen to draw. No one told me how to do it. I did it myself. Now, based on my practice, more and more farmers are doing the same.’ Daniel described how he modified the moldboard on the BBM which enables the seed to be covered automatically. This means that one less person has to be hired.

Women and men experiment with other technologies as well, for instance with varying dosages of pesticides and herbicides. Martha developed her own intercropping regime, though this failed. She has also built new poultry housing and experimented with feeding regimes. All four women make compost. Hannah has invented her own sowing container to distribute seed more evenly, she has made a wooden hoe, and she has spent considerable time on soil improvement. However, her expenditure on seed, fertilizer, and herbicide for growing improved wheat meant she could not employ anyone to help her in row planting. She secretly broadcast her seed at night so that the extension services would not see her. ‘I know I went totally against what is recommended,’ she said, ‘but I am sure I will have a good harvest.’ She accepted that her subversion would soon become apparent, but at least she had planted the way she felt necessary for her situation. It is not clear what would have happened had extension officers seen her, but clearly she felt it necessary to evade them.

This is in marked contrast to Soloman, who has a good relationship with the extension authorities despite frequently disregarding their advice. The four women innovators worry about censure; the four men do not.

### Sources of support to innovators

Our findings show that ‘supporters’ are critical in facilitating the work of innovators. At the same time, our innovators continually test and push against boundaries of socially acceptable behavior derived from deep structure, and thus challenge supporters. Table 2 lists sources of support and mentions by gender. Supporters include the extension services, neighbors, spouse, children, brothers, with single mentions of the kebele and uncles. Women innovators rely primarily on older and married children for support. Men draw, concurrently, on several support networks: the extension services, children, brothers, and, in all cases, the wife. Neighbors are rarely mentioned as sources of support.

**Table 2.** Sources of support.

	Women	Men
Extension services	X	XX
Neighbors		X
Wife		XXXX
Husband	X	
Children	XXX	XX
Brother		XX
Uncle	X	

#### Extension Services

Only one woman, Aysha, saw the extension services as supportive, no doubt because she is a model farmer. They have sent her on training courses, provided her with improved seeds, showed how to measure seed and fertilizer, and visit her regularly. This experience is unique to the women innovators. Even so, Aysha acknowledged that extension workers ‘are biased and distribute new varieties unfairly.’ Mimi spoke with open anger about the extension services saying they refuse to supply her with improved seed. She purchases improved seed from the market. Hannah and Martha do not discuss the extension services at all. Conversely, men respondents have a good relationship with extension officers, with Yonas viewing his relationship as critical. Soloman has the strongest relationship: ‘I have a good relationship with the DAs and people who distribute fertilizer and pesticide. The private research centers come here all the time to try out their new products.’

#### Neighbors

Apart from Yonas, innovators reported a fraught relationship with neighbors. Yonas said, ‘We farmers exchange new information all the time. If the issue is farm-related everyone wants to know about it. We deliberate pros and cons and ways to curb shortcomings. Some even encourage me to try out something. They follow my progress and give their opinion – mostly encouragement. But a few oppose me.’ All other men reported negative responses from neighbors. Soloman commented, ‘Farming is not something you hide from people’s eyes. It is out there for everyone to see. Whether you succeed or fail you cannot hide it. As for people’s opinion, I do not bother about what they say and I don’t need encouragement from anyone except the DAs and other experts able to give me meaningful advice.’ Women innovators reported active discouragement. According to Aysha, the model farmer, ‘neighbors gossip and insult me but I don’t listen. When I go to kebele meetings, they say look at her she is overreacting, they say did the kebele marry you that you go there all the time?’ Hannah discussed her plans with her friends prior to innovating, but said none supported her, rather they argued she was ‘crazy.’ One outcome was that she was unable to secure labor under debo, the reciprocal labor system. She added that ‘everyone’s eyes are on my plot. There are some who are hoping that I will fail. It is hard for a woman when she fails. People will talk about it for years. If it was a man no one will give it such attention.’

#### Spouses

The only married woman innovator receives full support from her husband, who has an off-farm business. All men innovators reported support from their wives. Soloman assumes full agreement to everything he plans; there is no discussion. Conversely, Yonas discusses everything with his wife and children. They are always supportive and share experiences they have heard about to encourage him. His wife says, ‘Go for it. If it is God’s will you will certainly succeed.’ Daniel, who has two wives, discusses his ideas with his youngest wife. His son now ‘decides everything’ for his older wife and farms with her. Ibrahim says these days, differently to the past, ‘I am taking it easy. I am learning to give space to my wife to decide also. And there are things you are required to consult your wife about.’ This is a consequence of changes to the law. Land, for instance, cannot be sold without the woman’s signature, a requirement Daniel chafed at.

#### Children

Children are significant supporters for three of the four innovator women. Aysha discusses her ideas with them. ‘They support me all the time.’ Martha’s children are the most supportive. ‘I consulted my children when I started the innovation. They were happy to try it out with me and worked day and night preparing the land. It was a difficult time.’ Whilst Hannah benefited at the beginning from her children who helped her prepare the land, they have now moved away apart from one son. She spoke repeatedly about how, now that he is older, he actively tries to block her innovations. ‘After my husband died, I decided everything until my son started to act as if he is the owner of the house. I struggle a lot. I even gave him land but he still tries to influence my decisions. He is doing this because he thinks he owns me. Despite this situation I am deciding for myself.’ All four men rely on their children’s labor, though Yonas spoke frequently of the importance of their schooling and providing good food. Ibrahim sent his son to be trained in the new technologies and was then trained by him. However, at first his other children did not support him. ‘They said our father is obsessed with these crazy ideas. They strongly disagreed.’ However, now, he reported rather grimly, ‘They started to zip their mouths when they saw the results. Now they would not agree if I stop cultivating this way.’

### Benefits from innovation

The respondents listed a variety of benefits from innovation as shown in Table 3.

**Table 3.** Benefits from the innovation.

	Women	Men
Improved respect in community	XXX	XXX
Strengthened self-respect	X	
More flexible mind		X
Improved house	XX	XX
Improved food security	X	XX
Children in school		XX
Improved income		XX
Rents more land		X
More livestock	XX	
Business diversification	X	X

Although neighbors rarely support innovators, they respect them when they succeed. Men respondents associated improved respect for being able to provide tangible evidence of their success, such as sending children to school, achieving food security, building a better house, and demonstrably having more money. Yonas said, ‘Since my yield is higher, the income I receive has increased. I have a large family and use the money to send my children to school. I am able to provide food for the family. This all happened because of the improved varieties and practices.’

Rather than discuss respect in terms of proxy indicators such as housing, women interpreted the word success more fluidly and in direct association with their sense of self. Although neighbors offer respect, this is highly contingent on innovatory women continuing to be ‘successful.’ ‘People who said bad things about me respect me now,’ said Hannah. She explained respect rose and fell in tandem with the progress of her innovatory practice. When she started row planting ‘respect was at the lower end’ but it started to improve as it brought results. When she was unable to continue row planting due to lack of funds respect ‘has probably gone down again. I have stopped noticing.’ Hannah attributed the core lack of respect she is accorded to her status as a widow. ‘Everyone wants to start rumors about me and some even want me to fail.’ Aysha reported that men dislike her because they believe she may ‘guide their wives,’ but that she is popular with women. Martha spoke of an increase in self-respect. Even though she failed in her wheat innovation ‘I learned a lot and it made me even more committed to make it. I know I am on the verge of success. I married off my daughter recently which made me proud because she did not end up in the streets. I’m in equib and edir groups. I have land and the capacity to get credit. Most of all my appetite for life has increased so much. I have hope.’ Mimi has become a member of equib and edir as a consequence of being recognized as an innovator.

## Discussion

This paper has examined how a small sample of women and men smallholders attempt to innovate with improved wheat seed, row planting, and the broad bed maker introduced through the Ethiopian agricultural extension system. The paper now returns to the tempered radicals conceptual framework to explore their strategies in the light of the data analysis presented above. Is it possible to define these innovators as tempered radicals? If so, the implications for supporting farmer-led innovation processes are then explored.

First, tempered radicals have a temper. Meyerson and Scully (1995, p. 586) define temper as both an ‘outburst of rage’ and ‘composure.’ Our innovators express temper in precisely these ways. They recounted furious arguments, and they also spoke with composure. The critical difference is that the men are buoyed by conceptualizing themselves as part of a global wave of social change. They feel part of the modern meta-narrative promoted by the extension services and some express contempt for farmers who do not change their practices. They closely associate the ability to adopt technologies with change in ‘mindsets.’ However, the women’s lack of self-identification with broader change and innovation processes is arguably a consequence of de facto exclusion by the extension services of almost all women from meetings and assistance. Neighbors and friends express strong negative voice in relation to both women and men innovators, but the consequences for women are more severe because they face social ostracization if they are deemed to go too far in transgressing the bounds of culturally acceptable feminine behavior. More than the men innovators, the women innovators use composure, expressed as skillful use of voice and silence, to push forward their agendas. Voice and silence are often considered opposites, yet silence is also a tool which can be a powerful way of achieving one’s goals (Morrison & Milliken, 2003). The findings suggest that our women innovators use their voice in limited ways and with caution. They do not lay claim to a role in meta-narratives. Their voice and self-determination are sometimes articulated through doing rather than speaking, as with the woman innovator who sowed at night.

Second, tempered radicals seek moderation. Moderation is necessary since innovators cannot risk being cast out of their communities. How, then, do they maintain the status of ‘outsiders within’ (Meyerson & Scully, 1995, p. 589)? What are their strategies for innovating yet belonging, and how successful are these strategies? Our women innovators are, a priori, recognized as outsiders within. They are widows, or divorcees (except for one case). As a consequence of their marital status, they experience marginalization, yet they occupy a recognized position as women heads of household. They use this position to subvert their marginalization in order to achieve their goals, for marginalization gives them much greater scope for setting their own agendas than most married women can contemplate. Beck (2009) considers that African women exploit the ambiguous space between norm and sanction to negotiate socially and culturally adequate gendered behavior. If so, our women innovators are constantly and consciously negotiating their status as innovators on the very boundary of what women are culturally deemed able to do. Beck (2009) continues by arguing that men frequently fear women who lead change processes because they associate this with a loss of personal power. In the case study, it would seem that women neighbors internalize male fears by watching women innovators from the margins, sometimes supporting them, sometimes mocking them. The remarks made by Alemu (2007) seem prescient here. He asserts that oral narratives can actively undermine the views of people – and specifically women – may hold of themselves. In order to innovate whilst avoiding sanction, the women innovators deliberately signal a desire to ‘belong,’ for example by joining indigenous societies. Acceptance into such structures, in turn, helps legitimatize their status as innovators – though this clearly is a fraught tension-laden process subject to continual challenge.

However, our men innovators do not need to consider how to negotiate their genderedness per se because their gender is the cultural norm. Men do not need to negotiate what they do, nor explain themselves (Alemu 2007; Beck, 2009). In the case study, the men innovators have an excellent relationship with the extension services, and more broadly see themselves as leading modernization processes. Men innovators are supported by externally driven meta-narratives, and locally validated deep structures (Alemu, 2007) which envision men as farmers and doers, whereas the women innovators must continually draw upon their inner resources, and children, to affirm and re-affirm themselves as individuals.

At the same time, men innovators are not entirely ‘within.’ They challenge local norms to a degree because they simultaneously engage with meta-narratives expressed through their interactions with extension services. Although they embrace the meta-narrative, they are also in critical dialog with it – a process remarked on by Østebø (2015) above in relation to gender. Men innovators challenge the ‘technically correct’ way of farming that they have been taught through introducing modifications they consider necessary in their agro-ecological environment. Their interactions do not necessarily endear them to neighbors, the majority of whom remain relatively unreached by the formal extension services, or who do not wish to interact. Male innovators thus occupy a curiously defiant liminal zone. As with women, their self-perception as innovators is their ultimate source of strength.

In the Introduction, we highlighted a learning model of innovation processes which is based on a Darwinian model of technologies moving towards fitness through a constant process of learning and adaptation (Douthwaite & Gummert, 2010). This is posited as a benign and optimistic model with constant evolution toward beneficial outcomes, provided the right institutional conditions are facilitated. ‘Over time, the evolutionary process changes the frequency and the variation of types of agents, strategies and artifacts as the population of fitter agents, strategies and artifacts increases in relation to others’ (Douthwaite & Gummert, 2010, p. 249). Our study casts doubt on this beneficial trajectory. The institutional environment is deeply configured by power relations articulated through the modus operandi of the extension services, and through socio-cultural norms which restrict the operation of women’s agency, and place milder limits on men’s agency. It is possible that the processes of innovation that the tempered radicals we describe have set in motion will be lost regardless of their potential fitness to the local context. Indeed, such is the power of the technology transfer model that it is almost impossible for it to perceive and capture the innovatory strategies of the people we initially called innovators, and who we now conclude are indeed tempered radicals.

## Conclusion

The rationale for this paper was to ask whether the concept of tempered radicals, developed originally to interrogate change processes in organizations, has validity in rural agricultural settings. We hypothesized that this concept might provide a useful lens through which the strategies of women and men farmers ‘doing things differently’ could be captured and analyzed. We also hypothesized that women and men may use broadly similar strategies, but that the implications for their personal standing in their communities, and the costs and benefits of using these strategies, may be different. We explored whether our eight innovators use the strategies ascribed to tempered radicals, and if so, whether the strategies are deployed in gendered ways. The conclusion must be that using the tempered radicals interpretative lens indeed allows us to recognize and interpret the strategies used by innovators in a novel way. New insights have emerged that are unique to the literature on agricultural innovators, and indeed enrich the tempered radicals canon of research literature through providing distinctive insights.

However, whilst the strategies deployed by the eight innovators discussed here conform to the tempered radicals framework, translating our insights into the development of strategies to identify and support grassroots innovators may be challenging. Our research and analysis show that innovation is happening and that there are windows of opportunities for supportive interventions (Leeuwis, 2013) but we also show that change is small, incremental, and faces resistance within communities regardless of the gender of the practitioner. The reasons for this are very different. Men draw legitimacy from ‘outside’; women draw legitimacy primarily from their own self-conceptualization. This self-conceptualization is rarely validated by the broader community.

One implication of our study is that strategies to support innovators, and women innovators, in particular, must be strongly context-specific and they must also be gender-sensitive. The literature review on Oromo cultural norms, and our findings, show that powerful institutional gender constraints to change processes exist and that they can be punitive for women. It will be necessary to understand these constraints through an examination of deep structures as well as visible expression of such structures. From here, strategies, in partnership with tempered radicals themselves, as well as local and also higher level institutions, to push the boundaries of what is deemed legitimate behavior for women and men can be developed. Developing ways to supporting change processes from within will help to strengthen women’s voice as well as promote agricultural innovation.

Our findings further show that single women, existing on the margins of their society, are more likely to be innovators because they have more decision-making power in relation to their own resources than women within male-headed households. There are two implications. First, there is clear scope for developing strategies to support women-headed households. Second, methodologies aiming to strengthen intra-household bargaining processes could be very useful. A number of household methodologies which perform this function should be considered (Farnworth et al., 2018).

## Notes

1 Petesch et al. (2018:35) describe ‘Economic dynamism’ as a competition over agriculture or NRM resources, infrastructure development, changes in the market orientation of smallholder farmers, processing technologies for key commodities, the relative percentages of buyers and sellers in local markets, and livelihood diversification, including on- and off-farm employment. Whereas ‘Gender gaps in assets and capacities’ include the percentage of girls completing primary school compared to boys, the extent to which women hold important leadership positions in local organizations, and norms about women’s freedom of movement.

2 The 2015–2017 national average is 27.6 quintal per hectare (FAOSTAT, http://www.fao.org/faostat/en/#data/QC).
